# Current distribution of Zostera seagrass meadows along the Bulgarian Black Sea coast (SW Black Sea, Bulgaria) (2010-2020)

**DOI:** 10.3897/BDJ.10.e78942

**Published:** 2022-02-09

**Authors:** Dimitar Berov, Stefania Klayn, Diana Deyanova, Ventzislav Karamfilov

**Affiliations:** 1 Institute of Biodiversity and Ecosystem Research, Bulgarian Academy of Sciences, Sofia, Bulgaria Institute of Biodiversity and Ecosystem Research, Bulgarian Academy of Sciences Sofia Bulgaria; 2 Department of Biological and Environmental Sciences, University of Gothenburg, Kristineberg, Fiskebäckskil, Sweden Department of Biological and Environmental Sciences, University of Gothenburg Kristineberg, Fiskebäckskil Sweden

**Keywords:** *Zosteramarina*, *Zosteranoltei*, Black Sea, seagrass meadows, EUNIS

## Abstract

**Background:**

The current distribution of *Zostera* spp. seagrass meadows along the Bulgarian Black Sea coast was studied. We used a combination of historical and recent observations of the habitat along the studied coastline. Remote sensing data (satellite images, sonar side-scans) was groundtruthed with georeferenced drop camera observations, scuba diving sampling and georeferenced scuba diving photo and video transects.

**New information:**

Тhe total area of the habitat type ‘MB548 - Black Sea seagrass meadows on lower infralittoral sands’ (EUNIS habitat type list 2019) in the study area is 916.9 ha, of which only 17.9 ha are in man-made sheltered environments (harbours). All seagrass meadows identified in 1978-79 were also located during the current survey, despite the increased eutrophication pressure and overall degradation of benthic habitats in the western Black Sea during the 1980s and early 1990s.

## Introduction

Seagrass meadows provide important ecosystem services as varied as nutrient cycling, habitat and food source for numerous fish, birds and invertebrates, coastal flood and erosion protection and are, thus, considered some of most valuable marine ecosystems (Nordlund et al. 2016, Nordlund et al. 2018). They are also part of the coastal "blue carbon" ecosystems that serve as a sink for organic carbon, sequestering it in their biomass and sediments (Fourqurean et al. 2012, Mazarrasa et al. 2018). Seagrasses have been in decline at a global scale throughout the 20^th^ century and at increasing rates since the 1990s due to coastal development, water quality deterioration and eutrophication, as well as climate change (Waycott et al. 2009).

There are seven species of vascular plants in the Black Sea, including four species of seagrasses (*Zosteramarina* L., *Zosteranoltei* Hornemann, *Cymodoceanodosa* (Ucria) Asch. and *Ruppiamaritima* L.) and three brackish water species (*Stuckeniapectinata* (L.) Börner, *Potamogetongramineus* L. and *Zannichelliapalustris* L.) (Kalugina-Gutnik 1975, Milchakova and Phillips 2003, Milchakova 2011, Karacuha and Okudan 2017). Seagrasses in the Black Sea form monospecific or mixed species communities, classified in five plant associations by Kalugina-Gutnik (1975). These include the *Zosteramarina* association, the *Zosteranoltei* association, the *Stuckeniapectinata* association, the *Ruppiaspiralis* association and the *Zannichelliapalustris*-*Zosteranoltei* association. Plant communities are structured by changes in water salinity and depth, as well as pollution and eutrophication levels (Kalugina-Gutnik 1975, Milchakova and Phillips 2003, Milchakova and Alexandrov 2011, Karamfilov et al. 2019).

The most extensive seagrass meadows in the Black Sea are found in its north-western part and along the Crimean coast (Ukraine and Russia), where they grow in large bays and gulfs, coastal lagoons and river mouths and deltas. That zone has the majority of seagrass habitats in the Black Sea, with an estimated area of 950 km^2^ (Milchakova and Phillips 2003, Milchakova and Alexandrov 2011). The Romanian Black Sea coast is relatively open and exposed to currents and winter storms, offering few suitable habitats for seagrasses. Romanian seagrass meadows declined significantly due to poor water quality in the 1980s and small ones are currently reported near Mangalia and Vama Veche (Surugiu 2008, Surugiu et al. 2021). Relatively small seagrass beds are present in bays along the whole Turkish Black Sea coast, particularly near Cape Sinop (Milchakova and Phillips 2003, Bilgin et al. 2007, Ersoy Karacuha et al. 2009, Karacuha and Okudan 2017).

To date, the only relatively complete survey of the presence and distribution of seagrasses along the Bulgarian Black Sea coast was carried out in the late 1970s (Petrova-Karajova 1982). It studied the depth structure and overall biomass of seagrasses within the Burgas Bay, setting up a historical baseline for the occurrence of this ecosystem in the area. No maps were created, leaving no spatial data or estimates of the overall area of the habitat from that period. The increased eutrophication pressure in the western Black Sea in the 1980s and the resulting decrease in water transparency, opportunistic green and red algal blooms, as well as bottom hypoxia, led to a degradation of phytobenthic communities - both *Cystoseira* macroalgal beds and *Zostera* seagrass meadows (Milchakova and Phillips 2003, Milchakova and Petrov 2003, Minicheva et al. 2013). This process affected the distribution of sensitive phytobenthic communities in the south-western part of the Basin, resulting in the disappearance of *Cystoseira* macroalgal beds from the Inner Burgas Bay (Berov et al. 2012).

Modern methods for mapping shallow-water marine habitats that combine remote sensing data from satellites, aerial orthophotography, drone photomosaic and side-scan sonar surveys, provide spatially extensive information that cannot be gathered with classical benthic sampling approaches (Phinn et al. 2008). The application of such methodologies, in combination with limited in-situ sampling and georeferenced visual/photo verification, provides opportunities for fast and reliable mapping of the spatial extent of seagrasses in large areas. Standardised methodologies that are based on free-for-use satellite image libraries (e.g. Google Earth), in combination with georeferenced underwater photography and video sampling, allow small teams of researchers to gather relevant data and fill data gaps in areas with little or no up-to-date information on the distribution of these valuable habitats (Roelfsema and Phinn 2009, Roelfsema et al. 2015).

## General description

### Purpose

The purpose of this study was to map the current distribution of Zostera spp. seagrass meadows along the Bulgarian Black Sea coast and to compare their current distribution (2010-2020) with historical data. We also aimed to set a baseline for future evaluations of changes in the spatial extent of this habitat type in the context of the Marine Strategy Framework Directive (MSFD) Bulgarian National Monitoring Programme (Descriptors 1, 5 and 6).

## Project description

### Title

Surveys and data analyses were undertaken in the framework of several research projects carried out by IBER-BAS in recent years. These include:

Enlargement of the Natura 2000 ecological network within the Bulgarian Black Sea sector. Contract 7976/04.04.2011 between MoEW and IO-BAS.

FP7 - Policy-oriented marine Environmental Research in the Southern EUropean Seas (Perseus); GA 287600.

FP7 - Towards COast to COast NETworks of marine protected areas (from the shore to the high and deep sea), coupled with sea-based wind energy potential (CoCoNET) GA 287844.

Balkan-Mediterranean 2014-2020- Regional cooperation for the transnational ecosystem sustainable development (Reconnect), Transnational Cooperation Programme Interreg V-B, co-funded by the European Union and national funds of the participating countries.

FEMA-MARE - Assessment and Mapping of MARINE Ecosystem Condition and Their Services in Bulgaria. Approved under programme BG03 Biodiversity and Ecosystems, financed by the EEA financial mechanism 2009-2014, Contract No. Д-33-87/27.08.2015.

MSFD National Monitoring Programme 2017.

“LTER - BG: Upgrading of the distributed scientific infrastructure Bulgarian Long-Term Ecosystem Research Network" under agreement D01-405/ 18.12.2020 with the Ministry of Education and Science (MES) of Bulgaria.

### Funding

''Enlargement of the Natura 2000 ecological network within the Bulgarian Black Sea sector". Contract 7976/04.04.2011 between MoEW and IO-BAS"; FP7 Perseus; FP7 Coconet; BalkanMed Reconnect; FEMA-MARE; MSFD National Monitoring Programme 2017; “LTER - BG: Upgrading of the distributed scientific infrastructure Bulgarian Long-Term Ecosystem Research Network" under agreement D01-405/ 18.12.2020 with the Ministry of Education and Science (MES) of Bulgaria.

## Sampling methods

### Study extent

The current distribution of *Zostera* spp. seagrass meadows along the Bulgarian Black Sea coast was studied - from Cape Sivriburun in the north, to the mouth of River Rezovska in the south. Our efforts were focused in the area with most abundant presence of seagrasses - Burgas Bay, but also included sites that provide favourable conditions for the development of seagrass beds.

### Sampling description

The mapping of the habitat extent was done using a combination of historical and recent observations of the habitat along the studied coastline following the methodological guidelines of Roelfsema and Phinn (2009) and Roelfsema et al. (2015).

We identified the presence of seagrass meadows in a certain area from the only published historical data from the late 1970s (Petrova-Karajova 1982), as well as from more recent studies and publications (Vassilev et al. 2005, Uzunova 2010, Dahl et al. 2016, Holmer et al. 2016, Karamfilov et al. 2019, Klayn and Karamfilov 2021), communication with local fishermen and divers and personal observations. Once the presence of seagrass meadows in a certain location was confirmed, we acquired the most recent and clear satellite images available in the Google Earth Pro libraries of the area. Georeferenced drone photomosaics were also used in certain locations, which provided more detailed spatial information and allowed us to follow the interannual variation in meadow size and extent. In certain locations, these methods were combined with high-resolution sonar side-scan mosaics, where the borders of the seagrass meadows were outlined, based on the clearly visible differences in texture of vegetated and non-vegetated sediments (Nikolopoulou et al. 2021).

The identified extent of seagrass meadows was verified in-situ by various methods. At a number of locations, scientific divers collected samples in predetermined locations following standard sampling procedures. Destructive samples were collected in order to help assess the impact of local eutrophication gradients on the ecological state of seagrass habitats, where *Z.noltei* was used as an indicator species (see Karamfilov et al. 2019 for details). This particular evaluation did not aim at obtaining a complete overview of the biometrics and biodiversity of all seagrass species in the studied areas. Whenever feasible, samples from the deeper *Z.marina*-dominated sections of the seagrass meadows were also collected.

Destructive samples were collected by a team of two SCUBA divers using a circular frame with cutting edges, with diameter of 31.5 cm and an area of 0.78 m^2^. The roots of the plants along the inner edge of the frame were carefully cut to a depth of 10–15 cm, making sure no roots, rhizomes or leaves from outside the sampling area fell into the sample. The plants were uprooted in a manner ensuring better removal of sand and mud, labelled and stored in a coolbox on board and transported to the laboratory where they were stored in a freezer at –20^◦^C. The wet weight biomass of leaves (aboveground biomass, AG W.W.) and of roots and rhizomes (belowground, BG W.W.) were measured on an electronic scale. The Leaf Area Index (LAI) of *Z.noltei* and *Z.marina* was measured following the methodology described in Short and Coles (2001).

In locations where the collection of destructive samples was not feasible, georeferenced digital photo and video transects were carried out, which allowed us to collect a large number of samples along the whole depth range of distribution of a given seagrass meadow (see Berov et al. 2018 for detailed description of applied methodology). Meadows with large spatial extent (e.g. Sarafovo), where scuba divers could not cover the whole extent of the habitat within a reasonable and safe number of dives, were groundtruthed with a drop camera deployed from a boat along predetermined points and transects (Fig. 2) (see Karamfilov et al. 2019). Images and video files were analysed by an experienced benthic ecologist who noted the presence of various species and identified habitat types in accordance with the EUNIS habitat classification scheme, as well as other attributes, such as sediment type and the coverage of the benthal by different organisms. The groundtruthing and mapping methods applied at each meadow were marked in the attribute table of the database under the column ‘map_method’. The date of the in-situ observation was noted in the column ‘dive_date’ and the date of the satellite image/side-scan used for the mapping is noted in the ‘Sat_date’ column.

For most seagrass meadows, we acquired satellite images that were more recent than the dates of the groundtruthing, allowing us to map their most recent spatial extent. In several cases, we could acquire a series of satellite images from different years, where the visible extent of the mapped meadows changed significantly over the period of a few years. In such locations, we mapped the seagrass meadow extent, based on the latest satellite image available. Thus the calculated seagrass area is valid for the data in the 'Sat_date' column of the attribute table.

Polygons with the actual area of the surveyed seagrass meadows were created manually in ArcMap 10.2. In-situ point data from samples and photo and video observations were overlaid on top of satellite images, photomosaics and side-scan mosaics and the visible boundary between vegetated and non-vegetated sea bottom was outlined (Fig. 2). Special care was taken not to misidentify shallow rocky reefs as seagrass beds, as they were often present in close vicinity of *Zostera* meadows and had similar colour characteristics on satellite photos (Figs 3, 4). In such locations, the correct mapping of the seagrass meadows requires detailed in-situ surveys, carried out by scuba divers applying georeferenced sampling and photo and video survey techniques that would allow the distinction between areas with segarass coverage from adjacent rocky reefs with macroalgae.

All survey methods and data analysis procedures, applied in this study, were initially tested, verified and approved in the seagrass meadows in Sozopol Bay (eLTER site Sozopol-Black Sea, https://deims.org/04c70bae-b13c-4df5-bbdb-dc2be9e9d411). The area has several seagrass meadows existing in a local pollution and eutrophication gradient with varying water quality, sediment properties and resulting community structure and ecological status (Holmer et al. 2016). The Zostera seagrass meadows in Sozopol Bay have been included in the long-term monitoring programme of this eLTER site since 2020.

A total of 1859 video and photo observations were filmed and analysed and 129 samples were collected and processed (Holmer et al. 2016, Anonymous 2018, Karamfilov et al. 2019) over the whole study period (2010-2020).

Above-ground biomass of *Z.marina*, sampled in seagrass meadows in Sozopol Bay in the summer season, varied between 28.3 g.m^-2^ and 60.8 g.m^-2^, while below-ground biomass was in the range between 25.8 g.m^-2^ and 66.4 g.m^-2^ (dry weight). *Z.marina* shoot densities were in the range between 295 sh.m^-2^ and 690 sh.m^-2^, leaf length was in the range betwen 110 mm and 719 mm and leaf area index values were between 4.2 m^2^.m^-2^ and 8.1 m^2^.m^-2^ .

Above-ground biomass of *Z.noltei* sampled in seagrass meadows in an eutrophication gradient in Burgas Bay (Karamfilov et al. 2019) in the summer season varied between 21.03 g.m^-2^ and 210.25 g.m^-2^, while below-ground biomass was in the range between 28.7 g.m^-2^ and 165.1 g.m^-2^ (dry weight). *Z.noltei* shoot densities were in the range between 747.1 sh.m^-2^ and 2246.5 sh.m^-2^, leaf length was in the range betwen 199.3 mm and 413.9 mm and leaf area index values were between 1.9 m^2^.m^-2^ and 3.8 m^2^.m^-2^.

**Figure 1.**
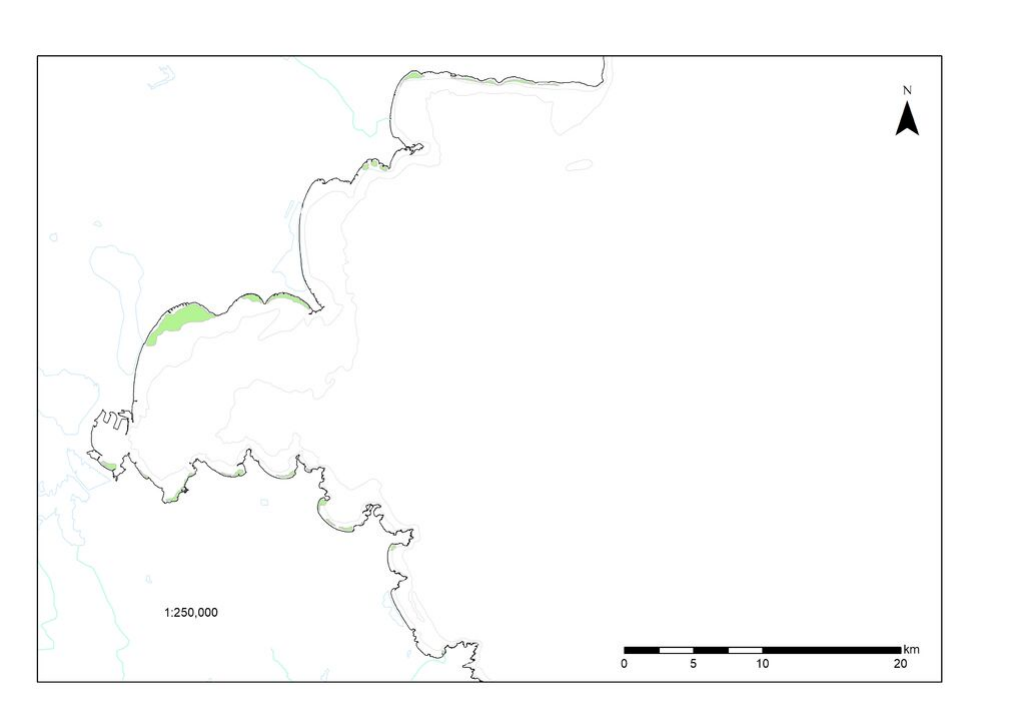
*Zostera* spp. seagrass meadows in Burgas Bay.

**Figure 2.**
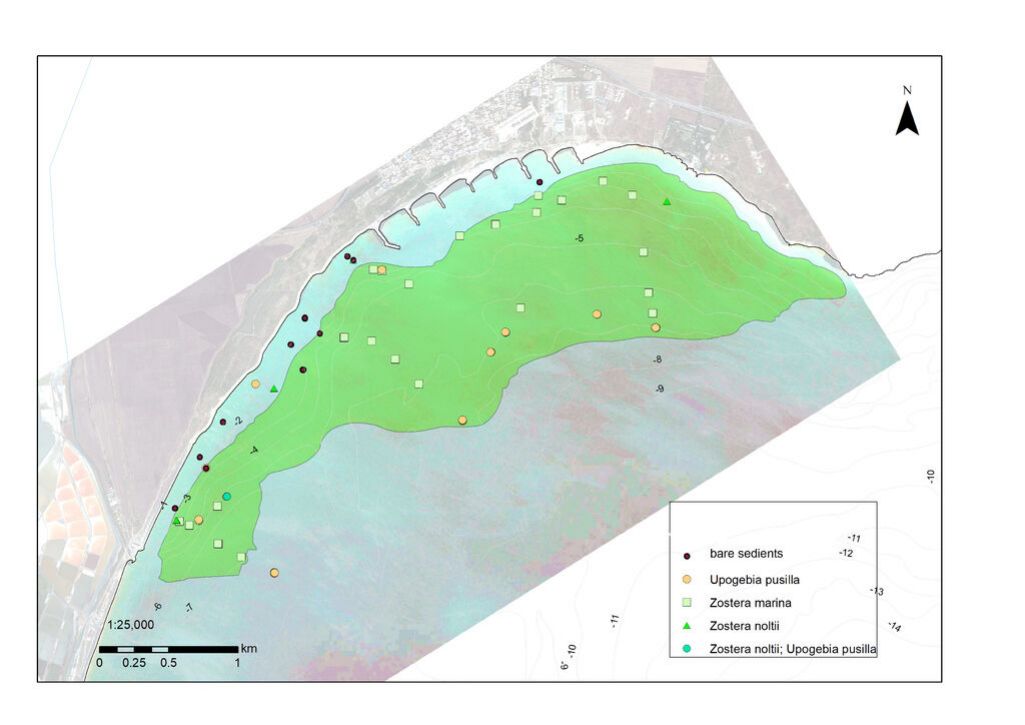
*Zostera* spp. seagrass meadow in front of Sarafovo, Inner Burgas Bay. In-situ data from drop camera deployments and scuba diving georeferenced photos are overlaid, with dominant species identified and labelled.

**Figure 3.**
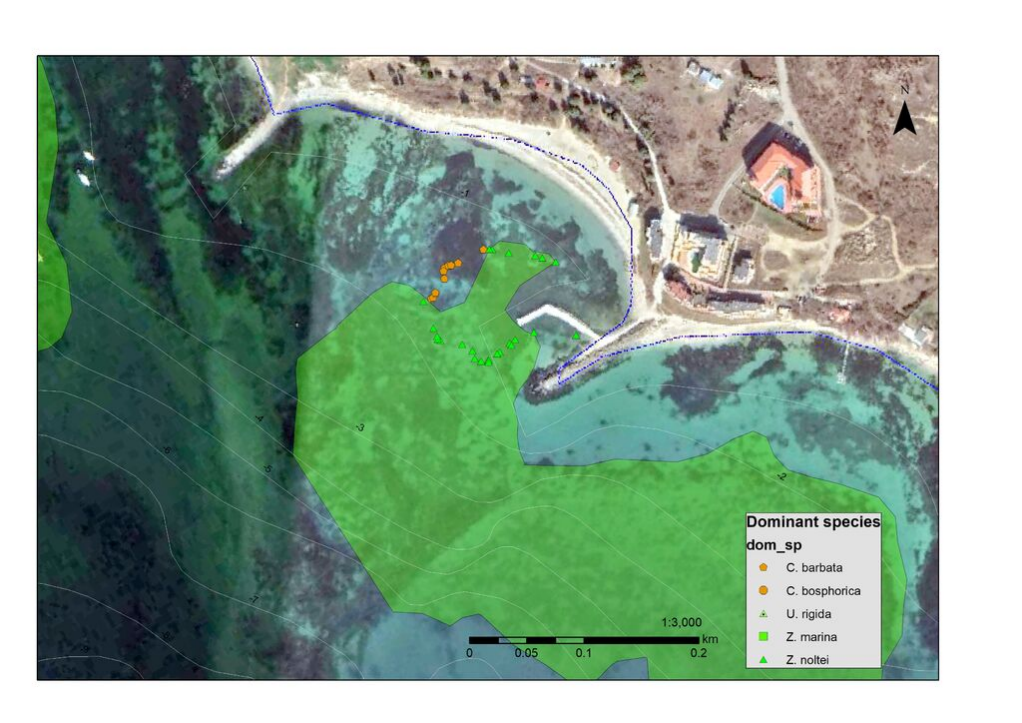
*Zostera* spp. seagrass meadow in front of Ravda with an adjacent rocky reef covered with *Gongolariabarbata* (Stackhouse) (sensu *Cystoseirabarbata* (Stackhouse)) with an overlay of data from a georeferenced photo scuba survey.

**Figure 4.**
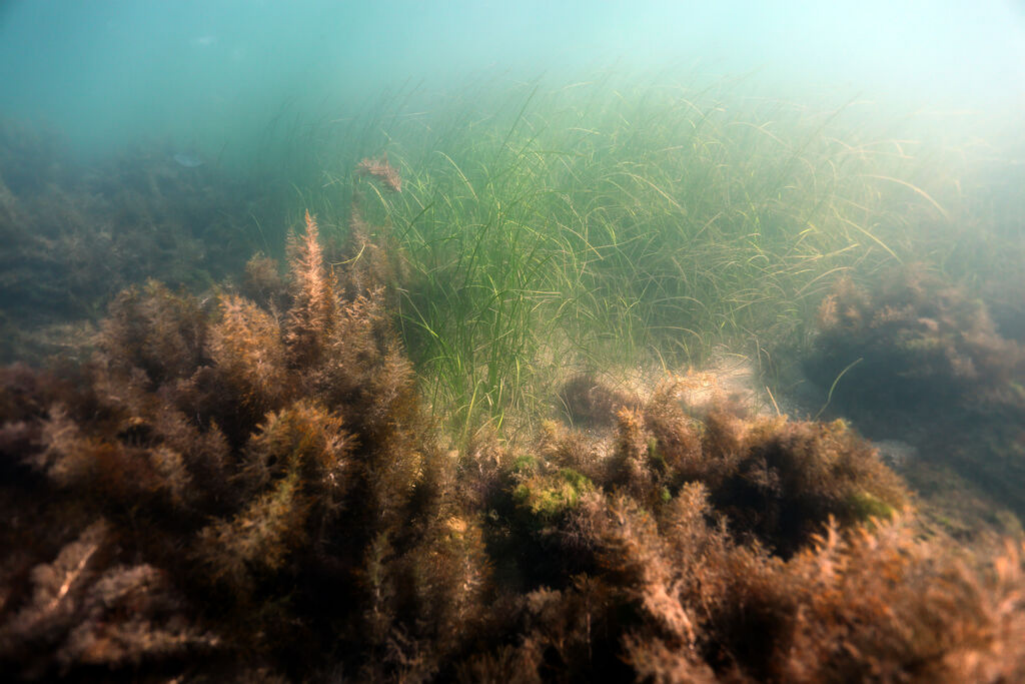
*Zosteramarina* Linnaeus on sandy bottoms growing next to a rocky reef covered with *Gongolariabarbata* (Stackhouse) (sensu Cystoseirabarbata (Stackhouse)) in a seagrass meadow in front of Ravda (depth ~ 2 m).

Тhe total area of the habitat type, classified in the EUNIS habitat types list (2019) as ‘MB548 - Black Sea seagrass meadows on lower infralittoral sands’ in the study area, is 916.9 ha, of which only 17.9 ha are in man-made sheltered environments (harbours) (Fig. 1). The seagrass bed in front of Burgas and Sarafovo represents 52% (411 ha) of the total area of this habitat in Bulgarian Black Sea waters. Other large meadows include those at Pomorie, Sunny Beach, Poda, Otmanli, Sozopol Bay and Sv. Vlas. All seagrass meadows identified by Petrova-Karajova (1982) in 1978-79 were also located during the current survey, despite the increased eutrophication pressures and overall degradation of benthic habitats in the western Black Sea during the 1980s and early 1990s.

### Quality control

Standard procedures for quality control in field sampling and laboratory sample processing were applied.

## Geographic coverage

### Description

Our studies covered the majority of the known *Zostera* seagrass meadows along the whole Bulgarian Black Sea coast, stretching from Cape Sivriburun in the north to Rezovska river mouth in the south.

## Temporal coverage

**Data range:** 2010-1-01 – 2020-12-31.

## Usage licence

### Usage licence

Creative Commons Public Domain Waiver (CC-Zero)

## Data resources

### Data package title

Current distribution of *Zostera* seagrass meadows along the SW coast of the Black Sea, Bulgaria.

### Resource link

https://www.seanoe.org/data/00684/79590/

### Alternative identifiers

https://doi.org/10.17882/79590

### Number of data sets

1

### Data set 1.

#### Data set name

Current distribution of *Zostera* seagrass meadows along the SW coast of the Black Sea, Bulgaria.

#### Data format

Shapefile

#### Number of columns

13

#### Download URL

https://www.seanoe.org/data/00684/79590/

**Data set 1.** 

Column label	Column description
FID	field ID.
Shape	shape field type.
NAME	name of seagrass meadow polygon.
map_method	Mapping methods applied for meadow mapping:satellite - seagrass meadows geographical extent mapping with satellite images; drone - seagrass meadows geographical extent mapping with georeferenced drone photomosaics; multibeam - seagrass meadows geographical extent mapping with multibeam survey; diving - verification through scuba diving methods; drop camera - verification with drop camera video/photo observations.
groundtruth	the identified meadow was groundtruthed: yes/no.
Sat_date	date of satellite image used in mapping.
area_ha	area in hectares.
insitudata	source of in-situ verification data.
Dive_date	date of in-situ diving for sampling.
Project	project name.
depthlimit	depth limit of seagrass meadow extend in metres.
type	type of seagrass meadow: deep - seagrass meadows developed in depths below 3-4 metres on exposed coastlines; shallow - seagrass meadows developed in depths starting from 1-2 metres on sheltered coastlines; harbour - seagrass meadows developed inside harbours and behind man-made structures.
EUNIS_2019	EUNIS 2019 habitat classification scheme habitat type.
